# Metabolic Dysfunction-Associated Steatotic Liver Disease and Polycystic Ovary Syndrome: A Complex Interplay

**DOI:** 10.3390/jcm13144243

**Published:** 2024-07-20

**Authors:** Konstantinos Arvanitakis, Elena Chatzikalil, Georgios Kalopitas, Dimitrios Patoulias, Djordje S. Popovic, Symeon Metallidis, Kalliopi Kotsa, Georgios Germanidis, Theocharis Koufakis

**Affiliations:** 1 Division of Gastroenterology and Hepatology, First Department of Internal Medicine, AHEPA University Hospital, Aristotle University of Thessaloniki, St. Kiriakidi 1, 54636 Thessaloniki, Greece; arvanitak@auth.gr (K.A.); gekalopi@auth.gr (G.K.); metallidissimeon@yahoo.gr (S.M.); georgiosgermanidis@gmail.com (G.G.); 2 Basic and Translational Research Unit, Special Unit for Biomedical Research and Education, School of Medicine, Faculty of Health Sciences, Aristotle University of Thessaloniki, 54636 Thessaloniki, Greece; 3 Athens Medical School, National and Kapodistrian University of Athens, 11527 Athens, Greece; ehatzikali@gmail.com; 4 Second Propaedeutic Department of Internal Medicine, Hippokration General Hospital, Aristotle University of Thessaloniki, 54642 Thessaloniki, Greece; dipatoulias@gmail.com; 5 Clinic for Endocrinology, Diabetes and Metabolic Disorders, Clinical Centre of Vojvodina, 21000 Novi Sad, Serbia; pitstop021@gmail.com; 6 Medical Faculty, University of Novi Sad, 21000 Novi Sad, Serbia; 7 Division of Endocrinology, First Department of Internal Medicine, AHEPA University Hospital, Aristotle University of Thessaloniki, St. Kiriakidi 1, 54636 Thessaloniki, Greece; kalmanthou@yahoo.gr

**Keywords:** MASLD, obesity, overweight, PCOS, infertility

## Abstract

Metabolic dysfunction-associated steatotic liver disease (MASLD) and polycystic ovary syndrome (PCOS) are prevalent conditions that have been correlated with infertility through overlapped pathophysiological mechanisms. MASLD is associated with metabolic syndrome and is considered among the major causes of chronic liver disease, while PCOS, which is characterized by ovulatory dysfunction and hyperandrogenism, is one of the leading causes of female infertility. The pathophysiological links between PCOS and MASLD have not yet been fully elucidated, with insulin resistance, hyperandrogenemia, obesity, and dyslipidemia being among the key pathways that contribute to liver lipid accumulation, inflammation, and fibrosis, aggravating liver dysfunction. On the other hand, MASLD exacerbates insulin resistance and metabolic dysregulation in women with PCOS, creating a vicious cycle of disease progression. Understanding the intricate relationship between MASLD and PCOS is crucial to improving clinical management, while collaborative efforts between different medical specialties are essential to optimize fertility and liver health outcomes in individuals with MASLD and PCOS. In this review, we summarize the complex interplay between MASLD and PCOS, highlighting the importance of increasing clinical attention to the prevention, diagnosis, and treatment of both entities.

## 1. Introduction

Metabolic dysfunction-associated steatotic liver disease (MASLD) is the new terminology proposed by three large multinational liver associations in 2023, in order to replace the term nonalcoholic fatty liver disease (NAFLD) [1]. MASLD, encompassing steatotic liver disease (SLD) and its more severe inflammatory form, metabolic dysfunction-associated steatohepatitis (MASH), has a prevalence that varies between 25.6% in women and 39.7% in men, and is the most frequent cause of chronic hepatopathy while also being a major cause of hepatic-related morbidity and mortality at a global level [2,3,4]. An excellent concordance rate has been demonstrated between MASLD and NAFLD, suggesting that 98% of patients diagnosed with NAFLD comply with MASLD criteria [5,6]. When metabolic indicators are absent, diagnoses are classified as cryptogenic SLD [6]. The pathophysiological mechanisms that lead to the development of MASLD have not yet been fully elucidated; however, fatty liver infiltration is considered to be the result of a “two-hit” process [7]. The “first hit” consists of acquired insulin resistance to fat deposition and hepatic steatosis caused by excess fatty acids, and the “second hit” is the result of cellular and molecular changes involving oxidative stress, lipid peroxidation, inflammation, and fibrosis [7]. Patients with MASLD are characterized by hepatic steatosis and at least one feature of the metabolic syndrome (MetS), mainly systemic hypertension, dyslipidemia, insulin resistance or hyperglycemia [8]. Insulin resistance, which is enhanced by elevated fat accumulation and upregulated differentiation of adipocytes, has a pivotal role, not only in the development of MASLD, but also in liver-related morbidity and extrahepatic complications [6,7]. Morbidity in patients with MASLD is primarily attributed to MASH, which is characterized by chronic hepatic inflammation and liver injury, progressively resulting in fibrosis and cirrhosis, with an increased risk of hepatocellular carcinoma (HCC) [9]. Recent conditional approval of resmetirom by the FDA, a thyroid hormone receptor-β-selective per os medication, is considered a major breakthrough in MASLD treatment, tailored for non-cirrhotic patients with MASH and moderate-to-advanced fibrosis [10,11].

Infertility is defined as failure to achieve a pregnancy after a year of regular unprotected intercourse and according to the World Health Organization, infertility affects around 15% of couples of reproductive age [12]. Infertility cases are divided into primary and secondary [13]. Primary infertility refers to cases in which a pregnancy has not been achieved according to the aforementioned definition, and secondary infertility includes cases in which at least one prior pregnancy has been achieved. In nearly 85% of the cases, an identifiable cause of infertility is present, while the rest of the cases are characterized as “unexplained infertility” [13]. Between the identifiable pathologies causing infertility (~85% of cases), ovulatory dysfunction, mainly due to polycystic ovary syndrome (PCOS), male factor infertility, and tubal disease, are considered the most common, while environmental factors, such as obesity, can enhance the pathophysiological processes leading to infertility [14,15]. PCOS represents 70% of cases with ovulatory dysfunction, with a prevalence of 10% in the general population [14,16]. Female infertility (FI) has a prevalence between 33% and 41% worldwide, and male infertility is individually responsible in approximately 30% of the cases and a contributing factor in a 20% of infertile couples [17]. FI is a complex disorder, caused by anatomical, autoimmune, hormonal and genetic abnormalities, and is highly associated with PCOS, endometriosis, and cardiometabolic and thyroid diseases [18]. Furthermore, the diagnosis of PCOS requires the presence of at least two of the Rotterdam criteria: clinical (e.g., hirsutism) or biochemical (e.g., elevated levels of free testosterone), hyperandrogenism, evidence of oligo-anovulation, and polycystic ovarian morphology appearing on ultrasound, and the exclusion of other relevant disorders, including non-classical congenital adrenal hyperplasia, Cushing syndrome, androgen-secreting tumors, hyperprolactinemia, thyroid diseases, and drug induced androgen excess [19]. Oligo-anovulation, or oligo-amenorrhea, is defined by the presence of fewer than 6–9 menstrual cycles in a year [19]. Polycystic-appearing ovarian morphology on ultrasound is characterized by an increased number (above 8–12) of intermediate follicles 8–12 mm in size, and by increased ovarian volume >10 cm^3^ for either ovary [19].

PCOS is the most common endocrine disorder in females of reproductive age, with a prevalence between 11% and 13% [20]. Furthermore, for the diagnosis of PCOS, the presence of at least two of the Rotterdam criteria is mandatory: clinical or biochemical hyperandrogenism, evidence of oligo-anovulation, polycystic ovary characteristics on ultrasound, and the exclusion of other relevant disorders [19]. PCOS is a multifactorial disease, characterized by and associated with metabolic, genetic, epigenetic, and environmental factors [21]. Insulin resistance is a potential contributor to the pathophysiology of PCOS, especially in hyperandrogenic women, independently of their body mass index (BMI) [22]. Persistent low-grade inflammation and high consumption of carbohydrates are also considered key contributors to pathophysiological alterations of PCOS [23]. Obesity, type 2 diabetes (T2D), and abnormal lipid metabolism, compose a complex metabolic profile in PCOS individuals [24,25]. Except from oligo/anovulatory infertility, PCOS-related implications include elevated risks of pregnancy complications and endometrial tumorigenesis [20]. Correlations with psychopathological conditions, including anxiety, depression, eating disorders, psychosexual dysfunction, and negative body image, have also been addressed [20]. PCOS management is adapted to the profile of each patient individually; however, in all cases the aims of therapeutic evaluation include reversing hyperandrogenism, inducing ovulation, regulating menstrual cycles and cardiometabolic clinical manifestations [26]. Many treatment choices are currently available, including oral contraception, anti-androgens, insulin sensitizers, ovulation inducers, and vitamin D supplements, which are in many cases used concomitant to each other, as none of them can individually fully eliminate the range of metabolic abnormalities in PCOS patients [27].

MASLD and PCOS are bidirectionally associated with insulin resistance, also overlapping in many other pathophysiological features [6,7,22]. Furthermore, evidence demonstrated that the presence of PCOS is an independent risk factor for MASLD development, increasing liver steatosis, and fibrosis in female patients [28,29]. The prevalence of MASLD lies between 34–70% in patients with coexisting PCOS diagnosis, which is highly elevated as compared with the general population (14–34%), even after adjusting for BMI [30]. The underlying pathophysiological mechanisms linking PCOS with MASLD are not completely understood, being mostly associated with insulin resistance and hyperandrogenism, however, metabolic dysregulation, dyslipidemia, obesity, and the hypothesis of low-grade chronic systemic inflammation, have also been proposed as culprits [5,31]. In addition to PCOS, MASLD has barely been associated with other causes of infertility, with a characteristic example being its recent correlation with male sexual dysfunction [32].

**Figure 1:**
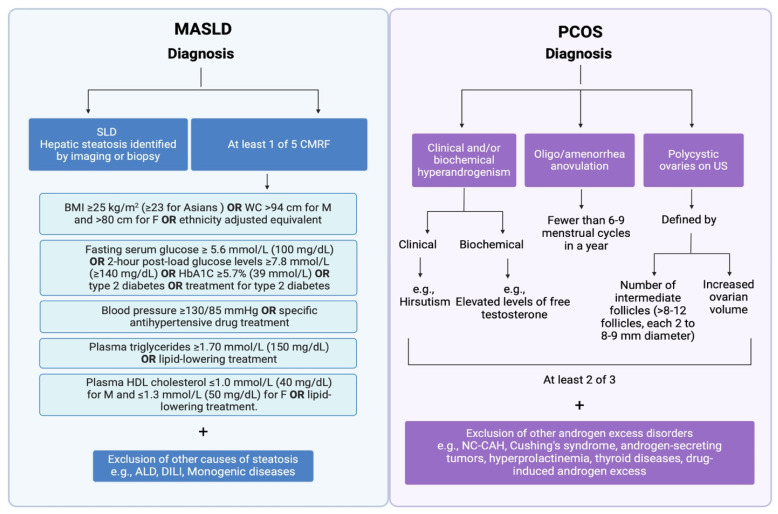
Newly established disease criteria for the diagnosis of MASLD and the Rotterdam criteria for the diagnosis of PCOS. MASLD is defined as hepatic steatosis combined with at least one out of five CMRF: (1) BMI ≥ 25 kg/m^2^ (≥23 kg/ m^2^ for Asians) or WC > 94 cm for males and >80 cm for females or ethnicity adjusted; (2) fasting serum glucose ≥ 5.6 mmol/L (100 mg/dL) or 2-h post-load glucose levels ≥ 7.8 mmol/L (≥140 mg/dL) or glycated hemoglobin ≥ 5.7% (39 mmol/L) or type 2 diabetes or treatment for type 2 diabetes; (3) blood pressure ≥ 130/85 mmHg or specific antihypertensive drug treatment; (4) plasma triglycerides ≥ 1.70 mmol/L (150 mg/dL) or lipid-lowering treatment; and (5) plasma high-density lipoprotein (HDL) cholesterol ≤ 1.0 mmol/L (40 mg/dL) for males and ≤1.3 mmol/L (50 mg/dL) for females or lipid-lowering treatment. Patients with SLD and at least one out of five CMRF are categorized as having MASLD when there are no other causes of steatosis present. According to the Rotterdam consensus, PCOS is defined by the presence of two out of three of the following criteria: oligo/amenorrhea or anovulation, hyperandrogenism (clinical and/or biochemical) and polycystic ovaries (≥12 follicles measuring 2–9 mm in diameter and/or an ovarian volume > 10 mL in at least one ovary). Created with BioRender.com, accessed on 7 June 2024. ALD; alcoholic liver disease; BMI: body mass index; CMRF: cardiometabolic risk factors; DILI: drug-induced liver injury; F: female; HbA1C: hemoglobin A1c; HDL: high density lipoprotein; M: male; MASLD: metabolic dysfunction-associated steatotic liver disease; NC-CAH: non-classical congenital adrenal hyperplasia; PCOS: polycystic ovary syndrome; SLD: steatotic liver disease; US: ultrasound; WC: waist circumference.

Our review provides a concise summary of the complex and multifaceted interplay between MASLD and infertility, delving into current knowledge on the underlying pathophysiological mechanisms that correlate MASLD with the development of PCOS, highlighting the importance of increased clinical awareness on timely diagnosis and therapy, but most importantly, on the prevention of PCOS in women with MASLD (Figure 1).

## 2. Pathophysiological Pathways Leading from PCOS to MASLD and Vice Versa

The pathophysiology of MASLD is complex and is associated with inflammation, lipotoxicity, and fibrosis, while its diagnosis requires the presence of positive criteria. These include evidence of hepatic steatosis detected by imaging or biopsy, prediabetes or T2D, a BMI above 25 kg/m^2^ or increased waist circumference, elevated blood pressure, increased triglyceride levels, low HDL levels, and an insulin-resistance index (HOMA-IR) higher than 2.5 or protein C levels greater than 2 mg/dL [33]. However, ruling out other chronic hepatic pathophysiological conditions or significant alcohol intake is not necessary for establishing MASLD diagnosis. Furthermore, given that MASLD affects more than 25% of the total population worldwide and has been associated with infertility, a deeper understanding of the underlying pathophysiological mechanisms that associate MASLD with infertility is of utmost importance [34]. The relationship between MASLD and female infertility has been at the forefront of medical research. PCOS, a pro-inflammatory condition accompanied with chronic low-grade inflammation that contributes to its pathogenesis, is the most common cause of anovulation and a major cause of infertility, characterized by a complex etiology and pathophysiology involving endocrine and reproductive disorders, including hyperandrogenism, insulin resistance, hyperinsulinemia, and hyperlipidemia [35]. In more detail, a recent study by Hong et al. found that women with PCOS exhibit a higher prevalence of MASLD and that this association is more prominent in obese women with higher insulin resistance. They also demonstrated that free testosterone level and free androgen index are independently associated with MASLD in women with PCOS, suggesting that hyperandrogenism contributes to the progression and development of MASLD in women with PCOS [36]. Along the same line, insulin resistance and hyperandrogenemia were found to be independent predictors of MASLD in Asian Indian women with PCOS [37]. Consequently, insulin resistance, hyperandrogenemia and hyperinsulinemia are among the principal pathogenic factors of the steroidogenic and metabolic dysregulation exhibited in women with MASLD and PCOS.

**Figure 2:**
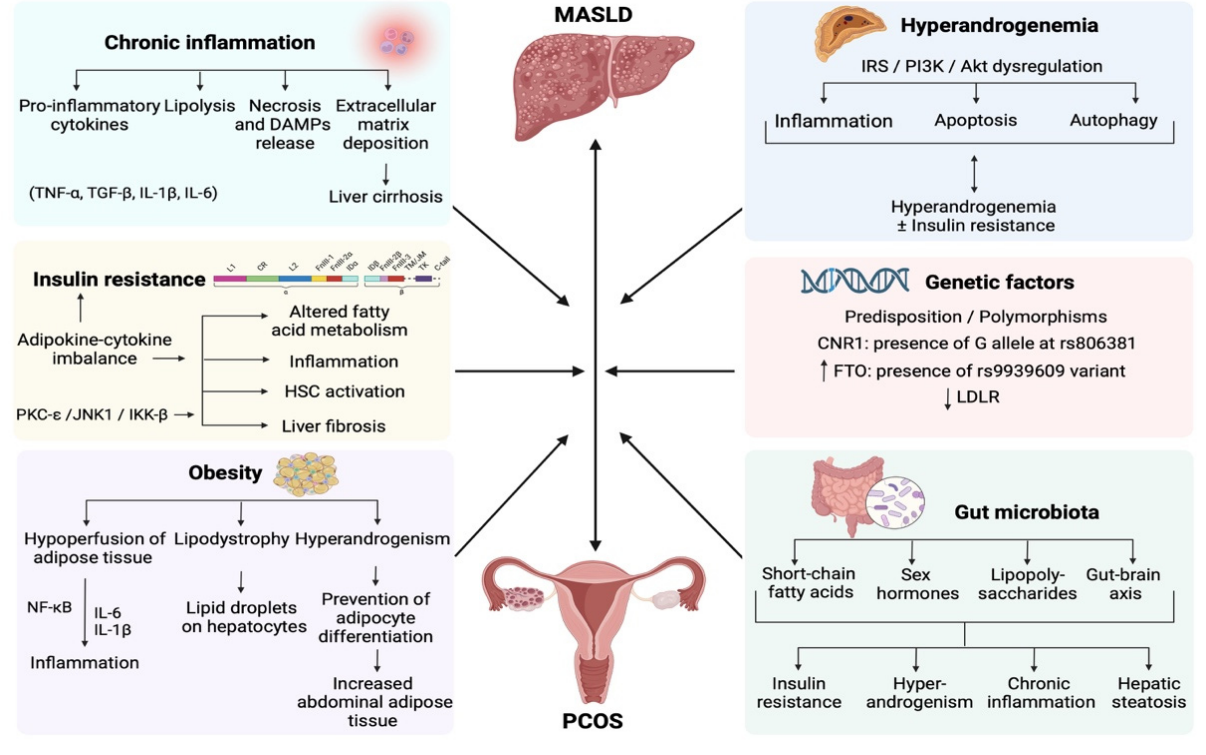
Schematic presentation of the pathophysiological mechanisms correlating MASLD with PCOS. Insulin resistance disturbs fatty acid and hepatic lipid metabolism, leading to a chronic low-grade inflammatory state and fibrosis, via the activation of HSCs. Obesity is associated with inflammation, lipodystrophy, and elevated androgen levels, while hyperandrogenemia is correlated with inflammation, apoptosis, and autophagy. Genetic factors, including predisposition and polymorphisms, play a crucial role in the development of MASLD and PCOS, while gut microbiota dysbiosis is associated with insulin resistance, hyperandrogenemia, inflammation, and steatosis. Chronic inflammation associated both with MASLD and PCOS, results in a necro-inflammatory pro-fibrinogenic hepatic environment, predisposing to liver cirrhosis. Created with BioRender.com. Abbreviations include: DAMPs: damage-associated molecular patterns; IKK-β: kappa-B kinase subunit beta; HSC: hepatic stellate cells; IL-1β: interleukin 1β; IL-6: interleukin 6; IRS-PI3K-Akt: Insulin receptor substance-phosphoinositide-3-kinase-Ak strain transforming; JNK1: c-Jun N-terminal kinases 1; MASLD: metabolic dysfunction-associated steatotic liver disease; NF-κB: nuclear factor kappa-light-chain-enhancer of activated B cells; PCOS: polycystic ovary syndrome; PKC-ε: protein kinase C epsilon; TNF-α: tumor necrosis factor-α; and TGF-β: transforming growth factor-β.

Another study investigating the prevalence and risk factors associated with hepatic steatosis in women with PCOS demonstrated that as compared to healthy controls, women with PCOS and MASLD had elevated BMI, waist circumference, triglycerides, total cholesterol, alanine and aspartate aminotransferases, and γ-glutamyltransferase, along with increased frequency of obesity and insulin resistance, concluding that since MetS is commonly related to hepatic steatosis, women with PCOS presenting with central adiposity and increased triglyceride levels should be screened for MASLD [38]. Moreover, a recent meta-analysis of 36 studies that evaluated the prevalence and risk factors of MASLD in patients with PCOS found a pooled MASLD prevalence of 43%, while BMI, waist circumference, alanine aminotransferase (ALT) and HOMA-IR values, levels of free androgen index, hyperandrogenism, and triglycerides were positively correlated with a significantly elevated risk of MASLD among PCOS patients. Furthermore, meta-regression showed that MASLD was correlated with the prevalence of MetS and HOMA-IR levels, the free androgen index and total testosterone levels [39]. The processes that lead women with PCOS to develop MASLD involve lipotoxicity, liver immune disturbances, along with hepatic and systemic insulin resistance, as well as intestinal dysbiosis, making MASLD a prototypic systemic metabolic disorder [40] (Figure 2).

### 2.1. Chronic Inflammation

Biomarkers of chronic inflammation play a significant role in understanding the pathophysiology and progression of both PCOS and MASLD. In PCOS, elevated levels of inflammatory biomarkers such as IL-6, TNF-α, and C-reactive protein are frequently observed. These biomarkers are indicative of systemic inflammation and are associated with insulin resistance, a hallmark of PCOS, contributing to metabolic dysregulation and cardiovascular risk. Likewise, in MASLD, chronic inflammation plays a pivotal role in disease progression, and biomarkers such as IL-6, TNF-α, and C-reactive protein reflect hepatic inflammation and are crucial indicators of disease severity, correlating with liver injury, fibrogenesis, and the development of cirrhosis [41,42]. The association of chronic inflammation biomarkers with both PCOS and NAFLD highlights their diagnostic and prognostic utility in assessing disease severity, monitoring treatment responses, and guiding therapeutic strategies aimed at mitigating inflammation and improving metabolic outcomes in affected individuals.

In more detail, high-calorie intake rich in saturated and trans-unsaturated fatty acids upregulates 3-hydroxy-3-methylglutaryl (HMG) CoA reductase and increases free cholesterol triggering interleukin (IL)-1β release from Kupffer cells. Furthermore, bacterial dysbiosis increases intestinal permeability and lipid absorption, as well as the translocation of gut-derived pathogen-associated molecular patterns (PAMPs) into the liver through the portal vein and into the systemic circulation leading to a pro-inflammatory hepatic state and the secretion of effector cytokines, including tumor necrosis factor-α (TNF-α), IL-1β, transforming growth factor-β (TGF-β), and IL-6. Insulin resistance, most commonly associated with T2D and obesity, promotes lipolysis of adipose tissue, leading to the breakdown of triacylglycerol stored in fat cells, releasing free fatty acids (FFAs) and glycerol into the circulation, which are subsequently transported and collected by other tissues to be utilized for β-oxidation and subsequent generation of adenosine triphosphate (ATP) [43]. Increased levels of FFAs and de novo lipogenesis potentiate fat accumulation in the liver, increasing the formation of reactive oxygen species (ROS) and stress that, along with the endoplasmic reticulum, activate the inflammasome, resulting in hepatocellular damage, apoptosis, the release of inflammatory mediators, and low-grade inflammatory conditions. These processes eventually lead to accumulation of lipid droplets in hepatocytes and steatosis, further potentiating cellular stress, lipotoxicity, and insulin resistance, by activating the kappa-B kinase subunit beta (IKK-β) [44]. Hepatocyte lipid deposition can result in their necrosis and the subsequent release of damage-associated molecular patterns (DAMPs) into the extracellular space, which can enhance inflammation in a Toll-like receptor 4 (TLR4)-dependent manner [45]. The aforementioned pathways increase endoplasmic reticulum stress, leading to activation of hepatic Kupffer cells that release pro-inflammatory cytokines including TGF-β, TNF-α, and IL-1β, alongside the activation of the profibrotic effect of hepatic stellate cells (HSCs), inducing fibrogenesis through extracellular matrix and collagen deposition, eventually aggravating metabolic dysfunction and liver fibrosis [46]. As a result, chronic inflammation ensues and HSCs can no longer participate in liver repair, leading to large-scale extracellular matrix deposition and fibrosis.

### 2.2. Insulin Resistance

Insulin resistance, which plays a pivotal role in the pathophysiology of T2D, hypertension, dyslipidemia and MetS, is characterized by reduced sensitivity of tissues to insulin, which is evident from the higher insulin concentrations necessary to reach optimal effect [47,48]. Furthermore, insulin resistance is strongly correlated with obesity, genetic predisposition, sedentary lifestyle, smoking, and stress, while due to the pivotal role that the liver plays in metabolism, a close relationship between MASLD and insulin resistance is established. Insulin resistance in MASLD arises from an imbalance between insulin sensitizing adipokines, such as adiponectin and leptin, and cytokines that promote insulin resistance like TNF-α. Decreased levels of adiponectin lead to altered fatty acid metabolism and long-term liver inflammation, while high serum leptin levels are found in individuals with MASLD and have been associated with HSC activation and liver fibrosis [49]. In more detail, impaired insulin ability to inhibit lipolysis processes in peripheral adipose tissue leads to increased levels of FFAs and elevated intrahepatic diacylglycerol concentrations, which are associated with activation of the protein kinase C epsilon (PKC-ε) and c-Jun N-terminal kinases 1 (JNK1) pathways, affecting insulin receptor substrates 1 and 2 and eventually aggravating insulin resistance and liver steatosis [50,51]. Furthermore, the activation of IKK-β in the setting of elevated oxidative stress by inflammatory cytokines such as TNF-α in MASLD, is associated with liver insulin resistance [51,52]. The increased anabolic functions mediated by hepatic insulin resistance result in elevated FFA concentrations, increased gluconeogenesis, disruption of insulin sensitivity in adipocytes, and increased lipogenesis.

Insulin resistance is linked to the buildup of visceral adipose tissue, which potentiates proinflammatory cytokines and adipokines, including IL-6, TNF-α, and leptin, enhancing lipolysis and stimulating the ovaries to produce androgens, leading to hyperandrogenemia [42]. The suppression of hormone-sensitive lipase in adipocytes due to insulin resistance, leads to increased lipolysis and a subsequent rise in FFA flow from adipose tissue to the liver, while hyperglycemia and hyperinsulinemia stimulate hepatic de novo lipogenesis by upregulating hepatic lipogenic transcription factors such as SREBP-1c and ChREBP, predisposing women with PCOS to the development of MASLD [53]. These factors enhance the activity of enzymes like glucokinase, fatty acid synthase, and acetyl-CoA carboxylase. Recently, proteins secreted by the liver known as hepatokines, have been discovered to play a vital role in glucose and lipid metabolism, thereby contributing to the pathogenesis of insulin resistance. It has also been demonstrated that the dysregulation of hepatokines, mainly fetuin-A, fibroblast growth factor-21 (FGF-21), and selenoprotein P1 (SEPP1), leads to the development of MetS, altered lipid metabolism, and increased oxidative stress in women with PCOS. Given the strong link between insulin resistance and PCOS, it is becoming increasingly evident that hepatokines, might play a role in the development of PCOS [54].

### 2.3. Obesity

Obesity is prevalent in women with PCOS, exacerbating MASLD in the setting of chronic low-grade inflammation and lipotoxicity. Premenopausal women with PCOS present a 2.5-fold greater risk of MASLD compared to controls, with increased BMI being the main cofactor [55]. Lipotoxicity occurs in the context of obesity, with a decreased capacity of adipose tissue to store excess energy, causing accumulation of lipid droplets in hepatocytes [56]. Hyperandrogenemia is considered one of the major causes of pathologically high abdominal visceral adiposity in women with PCOS [57]. In more detail, hyperandrogenism in PCOS causes weight gain due to the lipolytic action of androgens on adipocytes, releasing non-esterified fatty acids from visceral adipocytes, and preventing differentiation of adipocytes, as well as the formation of adipokines, leading to accumulation of abdominal adipose tissue [56]. Furthermore, in the setting of chronic low-grade inflammation associated with obesity, secreted adipokines exert adipogenic, pro-inflammatory, and fibrogenic functions, marked by hypertrophied adipocytes that cause interstitial vascular compression and hypoperfusion of adipose tissue, leading to hypoxia, stimulating activation of NF-κB and the pro-inflammatory cytokines IL-6 and IL-1β, which participate in the liver inflammatory response [58]. Indeed, in a rat model of hyperandrogenic PCOS, increased levels of serum inflammatory markers such as TNF-α and IL-1β, increased urocortin-1 mRNA expression along with the formation of a hepatic necrotic lesion, clearly demonstrating the onset of inflammation in the hyperandrogenic state of PCOS, indicating that it can lead to steatosis by altering the levels of hepatic inflammatory mediators and stress-related proteins [59].

### 2.4. Hyperandrogenemia

Hyperandrogenemia is also considered a common trait between PCOS and MASLD. Metabolic dysregulation is not only correlated with adipose tissue concentration and body weight in women with PCOS, but is also modulated by androgen excess [60]. Disrupted insulin signaling in polycystic ovaries leads to increased androgen secretion, decreased synthesis of sex binding hormones, and increased free androgens. A high free androgen index has been demonstrated to be associated with liver steatosis in women with PCOS, regardless of the presence of obesity and insulin resistance [61,62]. A study by Zhang et al. also demonstrated that in rats treated with insulin and/or human chorionic gonadotropin (hCG), there was a PPARα/β-Srebp1/2-Acc1 axis-mediated dysregulation between de novo lipogenesis and mitochondrial β-oxidation associated with hepatic steatosis, with hCG-induced hyperandrogenic rats exhibiting exacerbated hepatic inflammation [63]. Furthermore, inflammatory, apoptotic, and autophagic liver responses mediated by dysregulation of the IRS-PI3K-Akt signaling axis were highly correlated with hyperandrogenemia alone or combined with insulin resistance. Additionally, their study concluded that due to the fact that more pronounced liver steatosis, inflammatory response, and hepatocellular damage was observed in insulin- and hCG-induced PCOS-like rats, MASLD seen in individuals with PCOS is dependent on hyperandrogenism and insulin resistance, while hyperinsulinemia, hyperandrogenemia, and insulin resistance modify liver metabolism of lipids, hepatic function, and inflammatory response, with the effects of the individual conditions being distinct from each other. Along the same line, a meta-analysis demonstrated that the prevalence of MASLD increases in women with hyperandrogenic PCOS, as serum androgens, in addition to obesity and insulin resistance, were independent predictors of MASLD in women with PCOS [64].

### 2.5. Genetic Factors

Genetic predisposition and polymorphisms may play a pivotal role in the pathogenesis of MASLD in women with PCOS. In more detail, an analysis of variations in single nucleotide polymorphisms within the cannabinoid receptor 1 (*CNR1*) gene among women with PCOS revealed that the presence of the G allele at rs806381 was associated with an increased likelihood of MASLD, while genetic assays of adipose tissue from individuals with MASLD and PCOS indicated a potential correlation with diminished expression of the *LDLR* gene [65,66]. Abdominal obesity, dyslipidemia, and insulin resistance observed in women with PCOS and metabolic dysfunction have been associated with increased activity of the endocannabinoid system, while *CNR1* blockade has been shown to decrease serum aminotransferase levels and liver inflammation biomarkers in women with PCOS [67]. Furthermore, both MASLD and PCOS have been associated with increased obesity-associated gene expression (FTO), which is linked to increased oxidative stress, insulin resistance, and hepatic fat accumulation [68,69], with its common variant rs9939609 variant having been correlated with susceptibility to PCOS, potentially through its pronounced effect on BMI and insulin resistance [70]. However, more studies are required to establish the common genetic traits between MASLD and PCOS and their roles in pathogenesis and clinical outcomes.

Genome-wide association studies (GWAS) have revealed a notable association between PCOS and MASLD, suggesting a shared familial or genetic predisposition [41,71,72]. Individuals with a family history of PCOS are at an increased risk of developing MASLD, and vice versa, indicating that common hereditary factors may influence the pathogenesis of both conditions. In a bidirectional Mendelian randomization analysis, a recent study also demonstrated that genetically predicted MASLD is causally correlated with an increased risk of developing PCOS, with significant indirect causal effects via circulating levels of insulin and sex hormones [72]. This familial linkage may be attributed to inherited metabolic disturbances, such as insulin resistance, dyslipidemia, and obesity, which are prevalent in both PCOS and MASLD. Additionally, family members often share similar environmental and lifestyle factors, which can further contribute to the co-occurrence of these conditions [73,74]. The recognition of this family history association is critical for early screening and intervention strategies, allowing for the identification of at-risk individuals and the implementation of preventative measures to mitigate the progression of both PCOS and MASLD. This genetic interplay not only advances our understanding of the common etiological factors underlying these conditions, but also paves the way for targeted genetic screening and personalized therapeutic interventions. However, due to the scarcity of available data regarding the association of family history with PCOS and MASLD, future primary studies and randomized clinical trials are necessary in order to shed more light on the clinical importance of genetic interplay.

### 2.6. Gut Microbiota

Gut microbiota dysbiosis is another factor that plays an important role in the development and progression of MASLD and PCOS, impacting lipid and bile acid metabolism and absorption, insulin resistance, and regulation of the immune system [75]. In more detail, the gut microbiota of women with PCOS are associated with the presence of insulin resistance, hyperandrogenism, chronic low-grade inflammation, and hepatic steatosis, affecting the clinical presentation of PCOS through a variety of factors, including short chain fatty acids, lipopolysaccharides, sex hormones, and the gut-brain axis [76,77]. Hepatic steatosis-induced dysbiosis characterized by reduced IL-22 secretion is involved in increased lipopolysaccharide absorption and endotoxemia, short-chain fatty acid production, impaired bile acid metabolism, and abnormal secretion of brain-gut peptides, leading to a chronic inflammatory state through the proliferation of cytokines and mediators of inflammation such as TNF-α, IL-1, and IL-6 [78]. Furthermore, a high-calorie diet that increases intestinal permeability and alters its composition leads to lipotoxicity and increased deposition of liver fatty tissue, due to insulin resistance and inflammation [79]. Abnormal gut microbiota metabolism dysregulates intestinal endopeptides, cytokines, and inflammatory factors, leading to decreased levels of glucagon-like peptide-1, negatively impacting BMI and insulin resistance [80]. Therefore, restoring the gut microbiota may be a novel therapeutic option for patients with SLD and PCOS.

Recent studies have highlighted the potential benefits of symbiotic supplementation, which combines prebiotics and probiotics, on microbiome restoration and overall health in women with PCOS [81,82]. Symbiotics have been shown to modulate gut microbiota composition, enhancing the growth of beneficial bacteria and reducing pathogenic microorganisms. This rebalancing of the gut microbiome can improve metabolic and inflammatory parameters that are often disrupted in PCOS; for instance, symbiotic supplementation has been associated with improvements in insulin sensitivity, reduction in systemic inflammation, and favorable alterations in lipid profiles [83]. Similarly, another study reported significant improvements in plasma glucose and serum insulin levels in women with PCOS following an 8-week supplementation regimen with *L. casei*, *L. acidophilus*, *L. rhamnosus*, *L. bulgaricus*, *B. breve*, *B. longum*, and *Streptococcus thermophiles*, while Rashad et al. found that probiotic supplementation (*L. delbruekii* and *L. fermentum*) over 12 weeks led to a significant decrease of HOMA-IR levels and further improved the lipid profile [84,85]. These changes are crucial, as insulin resistance and chronic inflammation are central to the pathophysiology of PCOS. Additionally, the gut microbiome plays a role in regulating sex hormone levels, and its restoration may help in normalizing androgen levels, thus alleviating hyperandrogenic symptoms in women with PCOS [86]. Dietary changes aimed at increasing fiber intake and incorporating fermented foods can further support gut health and microbial diversity. Overall, incorporating symbiotics into the diet represents a promising therapeutic strategy to address the multifaceted symptoms of PCOS through gut microbiome modulation.

## 3. Summary

In conclusion, there is an undisputed metabolic influence in the pathophysiological processes that correlate MASLD with PCOS. PCOS is associated with MASLD through insulin resistance, chronic inflammation, genetic predisposition, hyperandrogenemia, obesity, and gut microbiota dysbiosis. Central visceral obesity associated with insulin resistance predisposes to the development of MASLD and PCOS, which in turn aggravates insulin resistance, leading to a vicious cycle. It is therefore crucial that physicians raise patient awareness regarding the benefits of an active lifestyle, careful weight management, and strict monitoring of glucose and lipid metabolism. It is also evident that hyperandrogenemia serves as a principal link between PCOS and MASLD, rendering the development of specific predictive models for the development of MASLD in women with PCOS of particular importance. Given the apparent high burden of MASLD, the implementation of screening programs could help identify MASLD and mitigate its consequences in a population where this condition has often been neglected. However, since current evidence has not yet clarified whether the presence of any of the individual features of PCOS is a cause, a consequence, or an epiphenomenon of MASLD, multicenter large-sample-size studies aiming to establish the temporal sequence of events alongside their causal relationship are necessary. Healthcare providers at the forefront of PCOS care should be highly vigilant and consider initiating, implementing and evaluating MASLD screening, specifically tailored for individuals with PCOS. Last but not least, close collaboration between medical specialties and an interdisciplinary approach involving metabolism, fertility, and hepatology specialists is necessary to improve conception and pregnancy outcomes in women with MASLD and PCOS.

## Data Availability

Not applicable.
